# Synergistic and antibiofilm activity of colistin combined with cinnamaldehyde and eugenol against colistin-resistant *Acinetobacter baumannii* clinical isolates

**DOI:** 10.1007/s10123-026-00848-7

**Published:** 2026-06-11

**Authors:** Abdulhamit Çalı, Rukiye Aslan, Kübra Fırtına Topcu, Cem Çelik

**Affiliations:** 1 Medical Laboratory Techniques, Vocational School of Health Services, Lokman Hekim University, Söğütözü. 2179 St., Çankaya, Ankara, 06510 Türkiye; 2 Pharmaceutical Microbiology, Department of Basic Pharmaceutical Sciences, Faculty of Pharmacy, Sivas Cumhuriyet University, Kayseri St. No:43, Sivas, 58140 Türkiye; 3 Department of Medical Microbiology, Faculty of Medicine, Sivas Cumhuriyet University, Kayseri St. No:43, Sivas, 58140 Türkiye

**Keywords:** *Acinetobacter baumannii*, Colistin resistance, Biofilms, Cinnamaldehyde, Eugenol, Synergy

## Abstract

**Objective:**

This study aimed to evaluate the effectiveness of combinations of colistin (COL) with cinnamaldehyde (CIN) or eugenol (EUG) against planktonic and biofilm forms of colistin-resistant clinical *Acinetobacter baumannii* isolates.

**Methods:**

Minimum inhibitory concentration (MIC) values of COL and essential oil components in 10 colistin-resistant clinical isolates were evaluated by the broth microdilution method. Interactions were classified by the fractional inhibitory concentration index (ΣFICI) using the checkerboard method. Biofilm formation was detected using the crystal violet method and the inhibitory effects of selected combination ratios on biofilm formation were evaluated in isolates showing synergy. In addition, the time-dependent effects of synergy combinations on 48-h pre-formed biofilm biomass were examined.

**Results:**

Median MIC values of COL, CIN, and EUG were 8 (4–32), 128 (128–128), and 512 (256–1024) µg/mL, respectively. In combination, the MIC of COL decreased to 1 (0.5–16) µg/mL with CIN and to 2 (0.5-8) µg/mL with EUG (*p* = 0.002). Synergy was observed in half of the isolates in both combinations (median ΣFICI: 0.532 and 0.469). Three isolates formed strong biofilms and seven formed moderate biofilms. Biofilm inhibition ranged from 1.1% to 50.5% for COL-CIN combination and from 11.7% to 80.0% for the COL-EUG combination. In 48-h pre-formed biofilms, the residual biomass at 24 h was significantly lower than that at 1 h for both combinations (*p* < 0.05).

**Conclusions:**

COL-CIN and COL-EUG combinations reduced MICs in colistin-resistant *A. baumannii* and decreased both biofilm formation and 48-h pre-formed biofilm biomass. These findings are promising for combination-based antibiofilm strategies.

**Supplementary Information:**

The online version contains supplementary material available at https://doi.org/10.1007/s10123-026-00848-7.

## Introduction

*Acinetobacter baumannii* is a leading cause of nosocomial infections, particularly in intensive care units, such as ventilator-associated pneumonia, catheter-related infections, and bacteremia (Yehya et al. 2025). This pathogen is considered among the global priority agents in the context of antimicrobial resistance (WHO 2024). Colistin-resistant (COL-resistant) *A. baumannii* infections can lead to clinical outcomes associated with high morbidity and mortality (Russo and Serapide 2025).

The ability of *A. baumannii* to form biofilms by adhering not only to biotic surfaces but also to various abiotic surfaces, especially medical devices such as ventilators and intravascular catheters, significantly contributes to the development of persistent infections and treatment failures (Pompilio et al. 2021; Scoffone et al. 2025). Due to the physical barrier provided by the extracellular matrix of the biofilm and the physiological heterogeneity within the biofilm, the effectiveness of antimicrobial agents can be significantly reduced (Gedefie et al. 2021). Therefore, treatments that demonstrate in-vitro efficacy against the planktonic form may be clinically insufficient for biofilm-associated infection. The limited effect of current antibiotics on biofilms further increases the need for targeted antibiofilm strategies (Pompilio et al. 2021).

Additionally, the increase in multidrug resistant strains of A. baumannii is driven by both intrinsic and acquired antibiotic resistance mechanisms (Novović and Jovčić 2023). COL is a last resort option for the treatment of *A. baumannii* infections because of the high rates of resistance to carbapenems (Novović and Jovčić 2023). However, the increase in resistance to COL, and especially the rise in cases of heteroresistance, is significantly complicating clinical management (Papazachariou et al. 2024). Mechanisms, such as changes in the structure of lipopolysaccharides, modifications in regulatory systems, and increased outer membrane permeability, play a role in the development of this resistance phenotype (Novović and Jovčić 2023; Papazachariou et al. 2024). Due to the inability to prevent the development of COL resistance, there is growing interest in combination studies aimed at increasing efficacy and limiting the development of resistance (Xie et al. 2022; Simbu et al. 2024; Islam et al. 2024). In this context, studies investigating antimicrobial combinations of conventional antibiotics with essential oil components (EOCs) have gained increasing attention in recent years (Ferrando et al. 2024; Simbu et al. 2024; Ibrahim and Mohammed 2025).

EOCs, such as cinnamaldehyde (CIN) and eugenol (EUG), are gaining increasing attention because of their membrane targeted effects on Gram-negative pathogens and their ability to suppress biofilm processes (Khwaza and Aderibigbe 2025). In particular, regarding *A. baumannii*, it has been reported that, in addition to increasing antibiotic susceptibility, CIN exhibits biofilm inhibition effects on multidrug resistant isolates, whereas EUG demonstrates antibiofilm activity even on 48-h pre-formed biofilms (Ganić et al. 2022; Choudhary et al. 2022). In addition, evidence regarding the systematic evaluation of synergy combinations of CIN and EUG with COL against both planktonic and biofilm forms in COL-resistant *A. baumannii* isolates is limited.

This study aimed to evaluate the effectiveness of combinations of COL with CIN and EUG against planktonic and biofilm forms of COL-resistant clinical *A. baumannii* isolates. The study focused on assessing changes in the minimum inhibitory concentration (MIC) of planktonic forms and synergy profiles using the checkerboard method. Additionally, it investigated the ability of selected combinations to inhibit biofilm formation and their time-dependent effects on 48-h pre-formed biofilm biomass.

## Materials and methods

### Study design, sample size, and isolates

This study was in vitro experimental research investigating the efficacy of COL combined with CIN and EUG against both planktonic and biofilm forms of COL-resistant *A. baumannii* isolates. The isolates were obtained from clinical samples sent to the microbiology laboratory for routine diagnosis from patients hospitalized at Sivas Cumhuriyet University Faculty of Medicine Application and Research Hospital. A total of 10 isolates were used in this study to ensure that only one isolate was obtained from each patient. COL resistance classification was performed by determining COL MIC values using broth microdilution, the European Committee on Antimicrobial Susceptibility Testing (EUCAST) reference method, and interpreting the results according to EUCAST clinical breakpoint values (EUCAST 2025).

### Isolation and identification

Clinical samples were inoculated onto one or more of the following media according to their characteristics: 5% sheep blood agar, EMB agar, and chocolate agar and incubated at 36 ± 1 °C for 24–48 h. At the end of incubation, isolates were preliminarily evaluated based on colony morphology, Gram staining, and conventional tests. Definitive identification was performed using matrix-assisted laser desorption ionization time-of-flight mass spectrometry (MALDI-TOF MS; Microflex LT, Bruker Daltonics, Germany) according to the manufacturer’s protocol. Identified isolates were stored in glycerol broth at −20 °C until used in the study.

### Essential oil components and antibiotic solutions

Stock solutions of COL (Sigma-Aldrich, St. Louis, MO, USA) were prepared in sterile distilled water in accordance with EUCAST recommendations and stored in aliquots at −20 °C. CIN (Bldpharm, Shanghai, China) and EUG (Bldpharm, Shanghai, China) were used as EOCs. These were dissolved in dimethyl sulfoxide (DMSO) to prepare stock concentrations of 51.2 mg/mL, and stored as aliquots at −20 °C. On each experimental day, working solutions were freshly prepared and diluted to target concentrations using cation-adjusted Mueller-Hinton broth (CAMHB). To eliminate solvent effects, a solvent control containing equivalent DMSO was used, and the final DMSO concentration in all wells was kept below 1% (v/v). The solvent control showed no visible effect on bacterial growth or biofilm biomass under the tested conditions.

### Microdilution method

The MIC values of COL, CIN, and EUG against the planktonic form were determined using the broth microdilution method in 96-well U-bottom microplates with CAMHB (EUCAST 2022). Two-fold serial dilutions were prepared at 64–0.125 µg/mL for COL and 1024–2 µg/mL for EOCs. A 0.5 McFarland suspension was prepared from isolates, diluted 1:100, and inoculated into the wells so that the final inoculum concentration was 5 × 10^5^ CFU/mL. Each microplate included growth, sterility, and solvent controls. The microplates were incubated at 37 °C for 18–24 h. The lowest concentration at which no visible growth was observed was recorded as the MIC. All experiments were performed in at least three replicates (Akıllı and İlki 2025).

### Checkerboard method

The interaction between COL, CIN, and EUG in A. baumannii isolates was evaluated using the checkerboard microdilution method (Molina Panadero et al. 2025). Two-fold serial dilutions were prepared in CAMHB from stock solutions of COL, CIN, and EUG. COL was dispensed along the horizontal axis and EOCs along the vertical axis of the microplates. For each isolate, the concentration range of both agents was adjusted from 1/16×MIC to 4×MIC based on the isolate’s individual MIC value. The final inoculum in the wells was 5 × 10^5^ CFU/mL, and the microplates were incubated at 37 °C for 18–24 h. The lowest combination concentrations with no growth were determined, and the FIC and ΣFICI values were calculated according to the following formulas:

$$\:{FIC}_{COL}=\:\frac{{MIC}_{COL,\:\:combination}}{{MIC}_{COL,\:alone}}$$

$$\:{FIC}_{EOC}=\:\frac{{MIC}_{EOC,combination}}{{MIC}_{EOC,alone}}$$

$$\:{\Sigma\:}\mathrm{F}\mathrm{I}\mathrm{C}\mathrm{I}=\:{FIC}_{COL}+{FIC}_{EOC}$$

Synergy was defined as an ΣFICI of ≤ 0.5, and additivity was defined as an ΣFICI of > 0.5 but < 1. Indifference was defined as an ΣFICI of ≥ 1 but < 4, whereas antagonism was defined as an ΣFICI of ≥ 4 (Lewis et al. 2002; Luna-De-Alba et al. 2024). For each isolate, the combination yielding the lowest ΣFICI was selected for summary analysis. If multiple pairs had the same ΣFICI, the pair with the lower COL concentration in combination was chosen. Each microplate included growth, sterility, and solvent controls. All experiments were performed in at least three replicates.

### Biofilm formation method

Biofilm formation was evaluated using the crystal violet microplate method described by Christensen (Christensen et al. 1985). Isolates were incubated overnight at 37 °C in tryptic soy broth (TSB) containing 1% glucose, adjusted to a turbidity of 0.5 McFarland, and inoculated into 96-well polystyrene microplates at 200 µL per well. The plates were incubated at 37 °C under static conditions for 48 h to allow biofilm formation. At the end of incubation, the wells were washed twice with phosphate-buffered saline (PBS) to remove planktonic cells. The biofilm layer was then stained with 0.1% crystal violet for 30 min. Excess stain was removed by washing twice with PBS. The bound stain was solubilized with 33% acetic acid for 30 min. The resulting color intensity was measured at 595 nm using a microplate reader (Biotek Synergy H1, USA) (Javadi et al. 2025). All experiments were performed in triplicate. Biofilm classification was based on the mean optical density (OD) value of the negative control (OD_NC_). Accordingly, OD ≤ OD_NC_ : non-biofilm producers; OD_NC_ < OD ≤ 2×OD_NC_ : weak biofilm producers; 2×OD_NC_ < OD ≤ 4×OD_NC_ : moderate biofilm producers; and OD > 4×OD_NC_ : strong biofilm producers (Chusri et al. 2012; Javadi et al. 2025).

### Biofilm inhibition method

The biofilm inhibition effect of COL-EOC combinations was evaluated only in isolate-combination pairs showing in the checkerboard assay, using the crystal violet microplate method in 96-well polystyrene microplates. The active substances were added to the wells before biofilm formation began, and the plates were incubated 37 °C for 48 h under static conditions. Based on the inhibitory concentrations (C_Comb_) of the combinations that showed a synergistic effect in the checkerboard analysis; COL, CIN, and EUG were tested at 0.5× and/or 1×C_Comb_ levels (0.5×COL_Comb_/0.5×EOC_Comb_; 0.5×COL_Comb_/1×EOC_Comb_; 1×COL_Comb_/0.5×EOC_Comb_). C_Comb_ was selected as the concentration of the combination that yielded the lowest ΣFICI in the checkerboard. At the end of incubation, the wells were washed twice with PBS to remove planktonic cells. The biofilm layer was stained with 0.1% crystal violet, and the bound stain was solubilized with 33% acetic acid. It was then measured at 595 nm using a microplate reader (Biotek Synergy H1, USA) (Çalı and Çelik 2024; Migliaccio et al. 2024). Each microplate included growth, sterility, and solvent controls. All experiments were performed in at least three replicates. Biofilm remaining and inhibition rates were calculated using the following formulas:

$$\begin{array}{c}Biofilm\:remaining\left(\%\right)=\\\frac{{(OD}_{Exposed}-{OD}_{Negative\:Control})}{{(OD}_{Positive\:Control}-{OD}_{Negative\:Control})}x100\end{array}$$

$$\:Biofilm\:inhibition\:\left(\%\right)=100-Biofilm\:remaining\:\left(\%\right)$$

### Time-dependent reduction in 48-h pre-formed biofilm biomass analysis

The time-dependent effect of synergistic COL-EOCs combinations on 48-h pre-formed biofilm biomass were evaluated using the crystal violet microplate method. Isolates were incubated overnight at 37 °C in TSB containing 1% glucose. Suspensions adjusted to 0.5 McFarland were added to 96-well polystyrene microplates and incubated under static condition at 37 °C for 48 h to allow biofilm formation. At the end of incubation, planktonic cells were removed, and the wells were gently washed with PBS. Concentrations of COL, CIN, and EUG that demonstrated synergistic effects were applied to the wells and incubated at 37 °C. Biofilm biomass was measured at 1, 4, 8, and 24 h using the crystal violet method. To monitor time-dependent changes, wells containing PBS without active substances were also observed at the same time-point. At each time-point, the supernatant was removed, and the wells were washed with PBS and dried. The biofilm was stained with 0.1% crystal violet for 30 min, excess stain was removed, and the bound stain was dissolved with 33% acetic acid for 30 min. Absorbance was measured at 595 nm using a microplate reader (Biotek Synergy H1, USA) (Hengzhuang et al. 2013; Chen et al. 2021). Each microplate included growth, sterility, and solvent controls. All experiments were performed in at least three replicates. Biofilm remaining and reduction rates were calculated using the following formulas:

$$\begin{array}{c}Biofilm\:remaining\left(\%\right)=\\\frac{{(OD}_{Exposed\:}{-OD}_{Negative\:Control})}{\left({OD}_{PBS}{-OD}_{Negative\:Control}\right)}x100\end{array}$$

$$\:Biofilm\:reduction\:\left(\%\right)=100-Biofilm\:remaining\:\left(\%\right)$$

### Statistical analysis

Data were presented as median (minimum-maximum) or n (%). The Wilcoxon signed-rank test was used to compare planktonic MIC values individually and in combination. Changes in COL MIC with checkerboard interaction were reported as fold-change. Time-dependent biofilm biomass measurements were normalized to the PBS control and expressed as a ratio, evaluated based on the -log_10_(OD_Exposed_/OD_PBS_) transformation. For differences between time-points, the Friedman test and Dunn’s multiple comparisons test were applied. *p* < 0.05 was considered statistically significant.

## Results

### Minimum inhibitory concentrations

The isolates were obtained from different clinical units and specimen types, predominantly respiratory samples from intensive care units. The isolate-level clinical source and routine antimicrobial susceptibility profile are presented in Supplementary Table S1.

The planktonic MIC values of COL and EOCs against 10 COL-resistant *A. baumannii* isolates were shown in Table 1. MIC values at the isolate level were presented in Supplementary Table S2. While a heterogeneous distribution of MIC values was observed among isolates for COL, similar activity was observed in all isolates for CIN. The MIC values of EUG were higher than those of CIN and showed a broader distribution.

**Table 1:** Planktonic MIC values of colistin and essential oil components against colistin resistant *A. baumannii* clinical isolates

Antibiotic and EOCs	*n*	MIC (µg mL^− 1^)		
		Median	Minimum	Maximum
Colistin	10	8	4	32
Cinnamaldehyde	10	128	128	128
Eugenol	10	512	256	1024

*MIC* minimum inhibitory concentration, *EOCs* essential oil components

### Synergistic interaction of colistin combinations

The combination of COL with CIN or EUG resulted in a decrease in the MICs required for bacterial growth inhibition compared to the single agent (Table 2). Based on the combinations that provided the lowest ΣFICI for each isolate, synergy was detected in five out of 10 isolates for both combinations. An additive effect was observed in the remaining isolates. An indifferent effect was observed only with the COL-CIN combination (Table 2). The isolate level FIC, ΣFICI values, and interaction classification were presented in Supplementary Table S3 and Table S4.

**Table 2:** Checkerboard assay summary for colistin combined with cinnamaldehyde or eugenol against colistin-resistant *A. baumannii* isolates (*n* = 10)

Parameter	CIN Combination Median (min-max)	*p*-value^†^	EUG Combination Median (min-max)	*p*-value^†^
Colistin MIC alone (µg/mL)	8 (4–32)	0.002*	8 (4–32)	0.002*
Colistin MIC in combination (µg/mL)	1 (0.5–16)		2 (0.5–8)	
EOC MIC alone (µg/mL)	128 (128–128)	0.002*	512 (256–1024)	0.002*
EOC MIC in combination (µg/mL)	48 (16–64)		64 (32–512)	
∑FICI	0.532 (0.375–1.000)	-	0.469 (0.25–0.75)	-
Colistin MIC fold-change	8 (2–16)	-	4 (2–16)	-
Interaction outcome (n, %)	Synergy 5 (50%); Additive 3 (30%); Indifferent 2 (20%)	-	Synergy 5 (50%); Additive 5 (50%)	-

† Wilcoxon matched-pairs signed-rank test (two-tailed, exact) comparing MIC alone vs. MIC in combination. *CIN* cinnamaldehyde, *EUG* eugenol, *EOC* essential oil components, *MIC * minimum inhibitory concentration, *FICI* fractional inhibitory concentration index

### Biofilm formation analysis

**Fig. 1:**
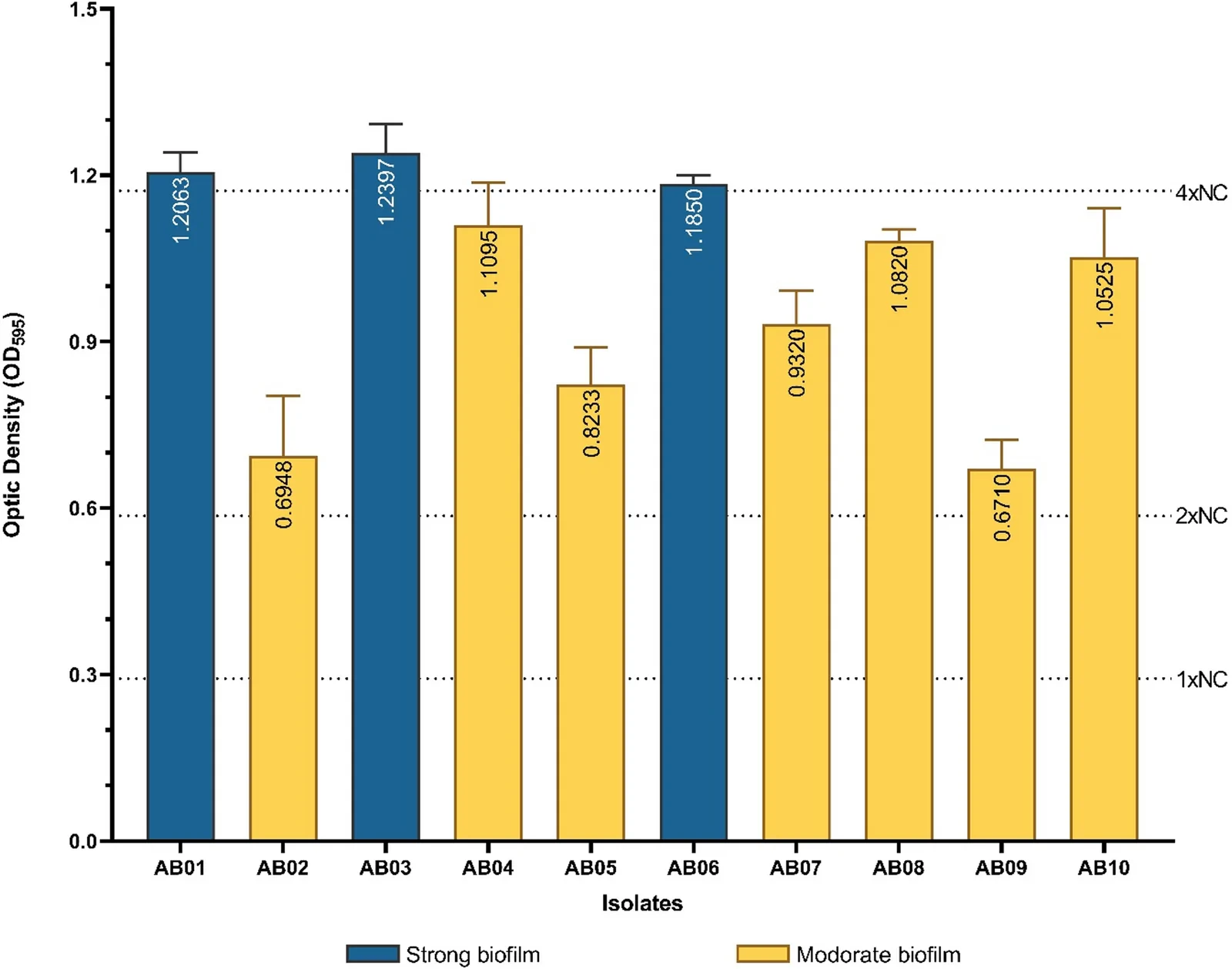
Biofilm formation activity of colistin-resistant *A. baumannii* isolates. Bars represent mean OD595 values with standard deviation. The dotted horizontal lines indicate the classification thresholds based on the negative control (OD_NC_; 2xOD_NC_; 4xOD_NC_)

All isolates formed measurable levels of biofilm as determined by the crystal violet microplate method. Based on the threshold values determined from the mean OD of the negative control, three isolates were classified as strong biofilm producers, while the remaining seven isolates were classified as moderate biofilm producers (Fig. 1, Supplementary Table S5).

### Effect of synergistic combinations on biofilm inhibition

**Fig. 2:**
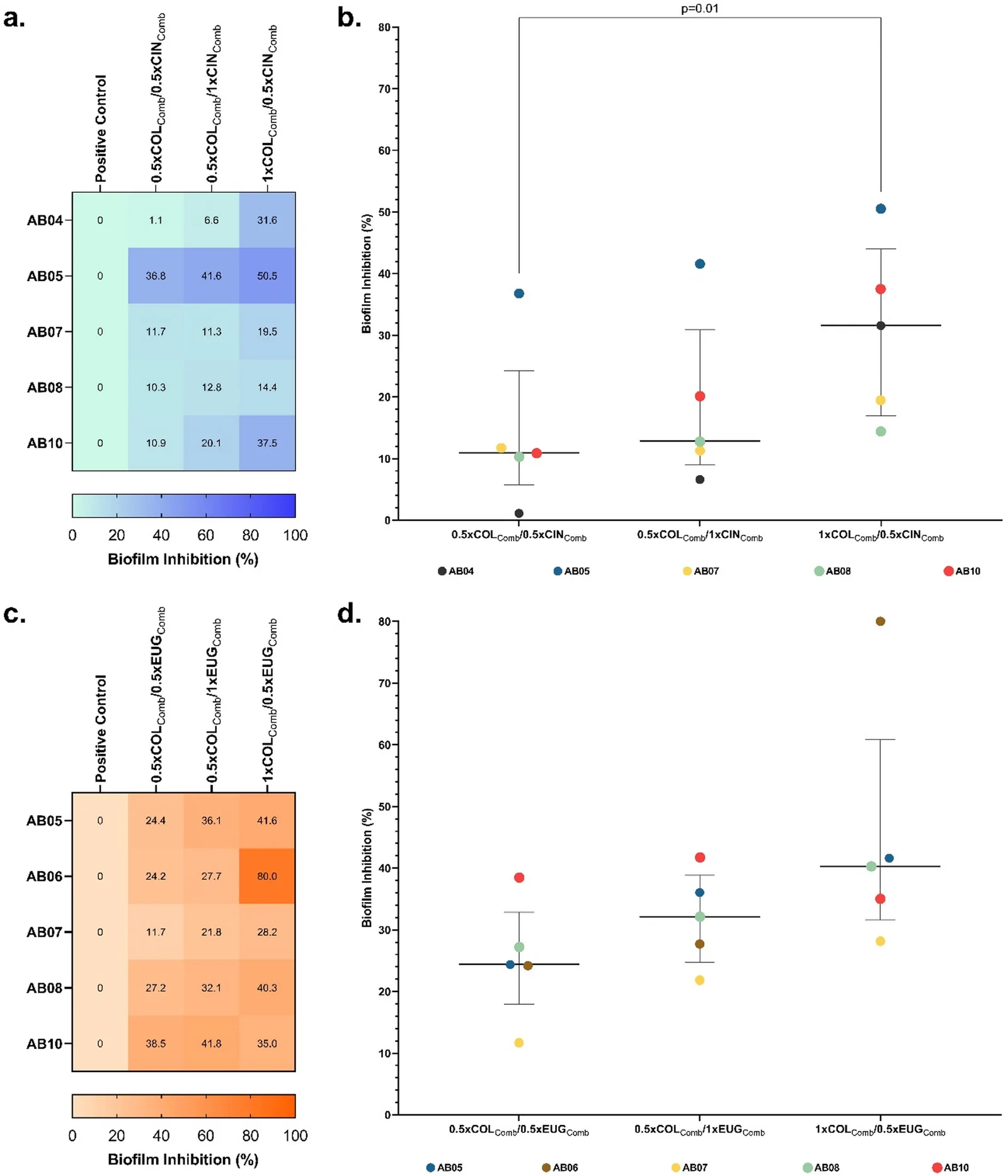
Biofilm inhibition of COL-EOC combinations. (**a-b**) Results for the COL-CIN combinations, (**c-d**) results for the COL-EUG combinations. Inhibition values are normalized relative to positive control and expressed as a percentage. Points represent the technical replicate mean for each isolate (*n* = 3). The horizontal line represents the median, and the error bars represent the interquartile range. Conditions were applied at 0.5× or 1× ratios based on the concentration pair (C_Comb_) that provided the lowest ΣFICI for each isolate in the checkerboard assay (COL_Comb_ and EOC_Comb_). Heatmaps (**a, c**) show the isolate-condition pattern, while scatter plots (**b, d**) show the distribution among isolates. Comparisons were performed using the Friedman test with Dunn’s correction. Significant p-values are indicated on the panel

In isolates where synergy was detected in the checkerboard assay, the potential of COL-CIN and COL-EUG combinations to inhibit biofilm formation was evaluated using the crystal violet microplate method, focusing on the concentration pairs yielding the lowest ΣFICI. In isolate-combination pairs where synergy was detected in the checkerboard assay, the potential of selected COL-CIN and COL-EUG combination ratios to inhibit biofilm formation was evaluated using the crystal violet microplate method. The isolate-combination pairs included in the analysis partially overlapped: AB05, AB07, AB08, and AB10 were evaluated for both COL-CIN and COL-EUG, whereas AB04 was evaluated only for COL-CIN and AB06 only for COL-EUG. Among the three strong biofilm-forming isolates, only AB06 was included in the biofilm inhibition assay because it showed a synergistic interaction with COL-EUG. The COL-CIN combination was found to be associated with biofilm inhibition, although this varied among isolates (Fig. 2, Supplementary Table S6). Inhibition values of the three ratios examined generally exhibited a distribution ranging from low to moderate levels (1.1%−50.5%). The most notable inhibition was observed at the 1×COL_Comb_/0.5×CIN_Comb_ condition in multiple isolates. In the COL-EUG combination, a heterogeneous distribution was also observed among the isolates, and inhibition values ranged over a wider interval (11.7%−80.0%) (Fig. 2). OD measurements at the isolate level and the corresponding biofilm remaining and inhibition percentages are provided in Supplementary Table S6.

### Time-dependent effect on 48-h pre-formed biofilm biomass

**Fig. 3:**
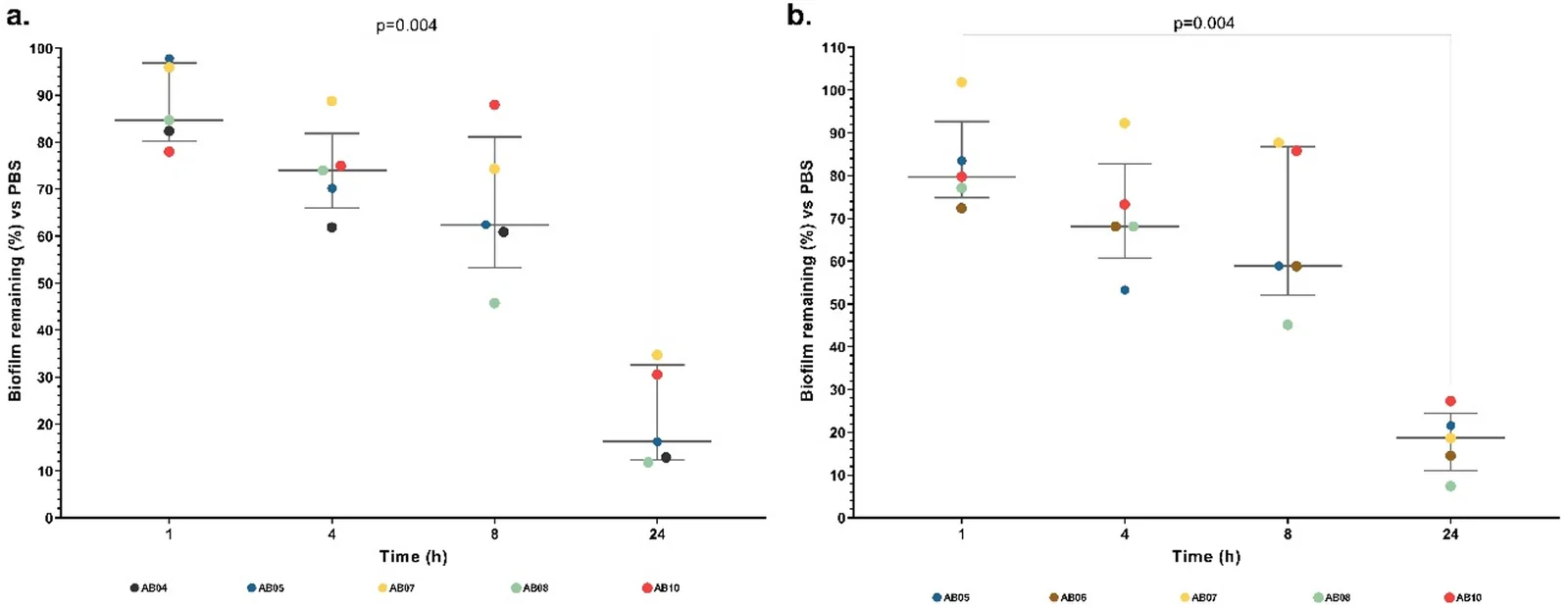
Time-dependent effect of COL combined with (**a**) CIN and (**b**) EUG on 48-h pre-formed biofilm. The y-axis shows biofilm remaining (%) normalized to the PBS control. Each point represents the technical replicate average of a single isolate. Horizontal lines indicate the median, and error bars indicate the interquartile range. Statistical comparisons were performed using the paired Friedman test based on the values of -log_10_(OD_COL−EOC/_OD_PBS_) and corrected with the Dunn multiple comparison test. Only p-value for significant comparisons is shown on the graph

The effects of COL-CIN and COL-EUG combinations applied to 48-h pre-formed biofilms at 1, 4, 8, and 24 h were evaluated using the crystal violet microplate method. Biofilm remaining values normalized to the PBS control varied among isolates over the time points. For both combinations, residual 48-h pre-formed biofilm biomass at 24 h was significantly lower than at 1 h (Fig. 3). While a gradual decrease was observed in some isolates over the time series, in others, the decline became evident at the later time point. At the isolate level, the ratios of the CIN and EUG combinations, mean OD values, PBS, and percentage of remaining biofilm calculations are shown in Supplementary Table S7 and S8.

## Discussion

In this study, the effectiveness of combinations of COL with CIN and EUG against both planktonic and biofilm forms of COL-resistant *A. baumannii* isolates was evaluated. It was determined that both combinations significantly reduced the inhibitory concentration at the planktonic level. In the checkerboard analysis, synergy was detected in half of the isolates, while an additive effect was observed in most of the remaining isolates. Additionally, all isolates formed moderate or strong biofilms, and sub-synergistic combinations inhibited biofilm formation. The gradual decrease observed over time in 48-h pre-formed biofilm biomass supports the potential of the COL-EOC combination approach for antibiofilm activity. The main contribution of this study is that it evaluates COL-based combinations not only against planktonic COL-resistant clinical *A. baumannii* isolates but also against biofilm formation and 48-h pre-formed biofilm biomass.

The distribution of planktonic MIC indicates heterogeneity among isolates for COL. The fact that EUG MIC values are higher and have a wider distribution compared to CIN MIC values suggests that the fundamental mechanisms of action and target interactions of EOCs may differ (Khwaza and Aderibigbe 2025). Nevertheless, consistent with previous studies, a significant reduction in MICs was observed when both EOCs were used in combination with COL (Vázquez-Ucha et al. 2020; Mireles et al. 2024). This supports the ability of CIN and EUG to enhance COL activity, even in the presence of COL resistance (Liu et al. 2023; Mireles et al. 2024).

According to the results of the checkerboard analysis, the median ΣFICI value for the CIN combination was 0.532 (0.375–1.000), while that for the EUG combination was 0.469 (0.250–0.750). These findings support that the combined use of COL with these two EOCs enhances each other’s effects against the isolates. The fact that synergy was not observed in all isolates can be explained by the emergence of COL resistance through multiple mechanisms. In *A. baumannii*, mechanisms such as changes in the structure of lipopolysaccharides, modifications in regulatory systems, and alterations in outer membrane permeability can play a role in COL resistance (Novović and Jovčić 2023; Papazachariou et al. 2024). Therefore, while the membrane-targeted effects of EOCs could theoretically increase COL efficacy, the combination of factors such as outer membrane composition and efflux activity among isolates may lead to varied responses. These mechanistic interpretations remain hypothetical, as no membrane permeability or gene-expression analyses were performed in the present study.

The biofilm form can reduce the effectiveness of antimicrobials due to the physical barrier created by the extracellular matrix and the physiological heterogeneity within the biofilm (Gedefie et al. 2021). In our study, the fact that three isolates produced strong and seven produced moderate levels of biofilm increases the clinical risk of *A. baumannii* infections. Based on the biofilm inhibition results, some of the strongest antibiofilm effects were observed under synergistic combination ratios that maintained relatively higher active concentrations within the tested range. Similar to the study conducted by Karumathil and colleagues, in our study it is particularly considered that EUG may contribute more strongly to antibiofilm activity during the early stages of biofilm formation (Karumathil et al. 2016).

In the analysis of reduction in 48-h pre-formed biofilm biomass, it was suggested that the effectiveness on 48-h pre-formed biofilm does not occur immediately, but rather through an effect that accumulates over time. This situation may be related to mechanisms such as the time required for agents to penetrate the biofilm matrix, the presence of metabolically active persistent cells within the biofilm, or anti-quorum sensing effects (Subathra et al. 2024).

These findings suggest that combinations of CIN and EUG with COL may represent a potential strategy for targeting both planktonic and biofilm forms in COL-resistant *A. baumannii* isolates. However, our results were obtained under in vitro conditions and with a limited number of isolates. Translation to clinical use remains uncertain because the achievable local or systemic concentrations, toxicity profile, and formulation feasibility of CIN and EUG were not evaluated. Further studies on the achievable tissue concentrations of EOCs, as well as their pharmacokinetics and pharmacodynamics, are warranted.

### Limitation

The main limitations of the study include the small sample size and the single-center design, which partially restrict generalizability. Second, synergy was assessed using the checkerboard assay rather than time-kill or MBC-based experiments. Therefore, the bactericidal or bacteriostatic nature of the COL-CIN and COL-EUG interactions could not be determined. Third, antibiofilm activity was evaluated mainly by crystal violet biomass measurements rather than viability-based methods. In addition, antibiofilm assays were limited to synergistic isolate-combination pairs and therefore did not include all isolates or all strong biofilm-forming isolates; thus, these antibiofilm findings should be interpreted as exploratory. Additionally, the study did not aim to conduct confirmatory investigations into the mechanism of action. Therefore, the findings presented in this study remain at the discussion level. Future studies are recommended to validate the findings in later, multicenter isolate panels, test the combination response under different experimental conditions, and evaluate the feasible formulation sizes of EOCs.

## Conclusion

In conclusion, the COL-CIN and COL-EUG combinations significantly reduced MIC values at the planktonic level in COL-resistant *A. baumannii* isolates and demonstrated synergy in half of the isolates. In this study, where all isolates formed biofilms, selected combination ratios inhibited biofilm formation to varying degrees in the tested synergistic isolate-combination pairs. Additionally, reductions in the biomass of 48-h pre-formed biofilm became more evident by 24 h.

Our findings suggest that the COL-EOC approach may represent a promising research avenue for COL-resistant *A. baumannii*, particularly in the context of biofilms. However, validation in larger sample sizes, dynamic synergy tests, biofilm viability-based measurements, and safety studies are required for clinical applicability. In this regard, our study provides a foundation for further research.

## Supplementary Information

Below is the link to the electronic supplementary material.

Supplementary Material 1 (DOCX 50.1 KB)

## Data Availability

The data underlying this article are available in the article and additional data will be provided on request.

## Abbreviations

CAMHB: cation-adjusted Mueller-Hinton broth
CIN: cinnamaldehyde
COL: colistin
DMSO: dimethyl sulfoxide
EUCAST: European Committee on Antimicrobial Susceptibility Testing
EUG: eugenol
EOC: essential oil components
FIC: fractional inhibitory concentration
FICI: fractional inhibitory concentration index
MALDI-TOF: matrix-assisted laser desorption ionization time-of-flight mass spectrometry
MIC: minimum inhibitory concentration
OD: optical density
PBS: phosphate-buffered saline
TSB: tryptic soy broth
MBC: minimum bactericidal concentration
