# Supplementary material for: Synergistic and antibiofilm activity of colistin combined with cinnamaldehyde and eugenol against colistin-resistant Acinetobacter baumannii clinical isolates
Source: Int Microbiol. 2026 Jun 11;29(7):1319–28. doi: 10.1007/s10123-026-00848-7 (PMC13653846; doi:10.1007/s10123-026-00848-7)
Supplement: Supplementary file 1 [file 10123_2026_848_MOESM1_ESM.docx]

**Synergistic and Antibiofilm Activity of Colistin Combined with Cinnamaldehyde and Eugenol against Colistin-Resistant *Acinetobacter baumannii* Clinical Isolates**

**Tables**

Table S1. Clinical source and antimicrobial susceptibility profile of colistin-resistant A. baumannii isolates.

Table S2. MICs of colistin, cinnamaldehyde, and eugenol for individual colistin-resistant *A. baumannii* isolates (AB01-AB10).

Table S3. Isolate-level checkerboard results for the colistin-cinnamaldehyde combination against colistin-resistant *A. baumannii*.

Table S4. Isolate-level checkerboard results for the colistin-eugenol combination against colistin-resistant *A. baumannii*.

Table S5. Quantitative biofilm biomass and biofilm classification of colistin-resistant *A. baumannii.*

Table S6. Isolate-level biofilm inhibition by colistin-cinnamaldehyde and colistin-eugenol combinations in colistin-resistant *A. baumannii*.

Table S7. Time-dependent biofilm remaining of colistin-cinnamaldehyde combination relative to PBS.

Table S8. Time-dependent biofilm remaining of colistin-eugenol combination relative to PBS.

**Table S1.** Clinical source and antimicrobial susceptibility profile of colistin-resistant *A. baumannii* isolates.

| **Isolates** | **Ward** | **Specimen type** | **Isolation date** | **AMK** | **SAM** | **CIP** | **COL** | **GEN** | **IPM** | **LVX** | **MEM** | **SXT** | **TOB** |
| --- | --- | --- | --- | --- | --- | --- | --- | --- | --- | --- | --- | --- | --- |
| AB01 | ICU | Sputum | 18.10.2024 | R | R | R | R | R | R | R | R | R | NT |
| AB02 | ICU | Sputum | 30.09.2024 | R | R | R | R | R | R | R | R | R | NT |
| AB03 | Neurosurgery ICU | Sputum | 03.12.2024 | R | R | R | R | R | R | R | R | R | NT |
| AB04 | Internal medicine ICU | Aspirate | 03.10.2024 | R | R | R | R | R | R | R | R | R | NT |
| AB05 | Physical therapy and rehabilitation unit | Wound swab | 20.12.2024 | R | R | R | R | R | R | R | R | R | NT |
| AB06 | Emergency | Respiratory secretion | 23.10.2024 | R | R | R | R | R | R | R | R | R | NT |
| AB07 | ICU | Sputum | 22.12.2024 | R | R | R | R | R | R | R | R | R | NT |
| AB08 | Internal medicine ICU | Sputum | 24.10.2024 | R | I / susceptible, increased exposure | R | R | R | R | R | R | R | NT |
| AB09 | Emergency | Urine | 09.01.2025 | R | R | R | R | R | R | R | R | R | R |
| AB10 | Internal medicine ICU | Sputum | 28.11.2024 | R | R | R | R | R | R | R | R | R | NT |
| Abbreviations: ICU, intensive care unit; AMK: amikacin; SAM: ampicillin-sulbactam; CIP: ciprofloxacin; COL: colistin; GEN: gentamicin; IPM: imipenem; LVX: levofloxacin; MEM: meropenem; SXT: trimethoprim-sulfamethoxazole; TOB: tobramycin; R, resistant; I, susceptible, increased exposure according to EUCAST terminology; NT, not tested. | | | | | | | | | | | | | |

**Table S2.** MICs of colistin, cinnamaldehyde, and eugenol for individual colistin-resistant *A. baumannii* isolates (AB01-AB10).

| **Isolates** | **MIC_COL_ (µg mL^-1^)** | **MIC_CIN_ (µg mL^-1^)** | **MIC_EUG_ (µg mL^-1^)** |
| --- | --- | --- | --- |
| AB01 | 4 | 128 | 512 |
| AB02 | 32 | 128 | 1024 |
| AB03 | 16 | 128 | 512 |
| AB04 | 16 | 128 | 512 |
| AB05 | 4 | 128 | 512 |
| AB06 | 8 | 128 | 512 |
| AB07 | 4 | 128 | 256 |
| AB08 | 8 | 128 | 512 |
| AB09 | 8 | 128 | 256 |
| AB10 | 4 | 128 | 512 |

*MIC: minimum inhibitory concentration; COL: colistin; CIN: cinnamaldehyde; EUG: eugenol.*

**Table S3.** Isolate-level checkerboard results for the colistin-cinnamaldehyde combination against colistin-resistant *A. baumannii*.

| **Isolates** | **Alone MIC_COL_** | **Alone MIC_CIN_** | **Combination MIC_COL_** | **Combination MIC_CIN_** | **FIC_COL_** | **FIC_CIN_** | **∑FICI** | **Description** | **Colistin MIC fold-change** |
| --- | --- | --- | --- | --- | --- | --- | --- | --- | --- |
| AB01 | 4 | 128 | 2 | 64 | 0.500 | 0.500 | 1.000 | Indifferent | 2 |
| AB02 | 32 | 128 | 16 | 64 | 0.500 | 0.500 | 1.000 | Indifferent | 2 |
| AB03 | 16 | 128 | 2 | 64 | 0.125 | 0.500 | 0.625 | Additive | 8 |
| AB04 | 16 | 128 | 2 | 32 | 0.125 | 0.250 | 0.375 | Synergy | 8 |
| AB05 | 4 | 128 | 0.5 | 32 | 0.125 | 0.250 | 0.375 | Synergy | 8 |
| AB06 | 8 | 128 | 0.5 | 64 | 0.063 | 0.500 | 0.563 | Additive | 16 |
| AB07 | 4 | 128 | 1 | 16 | 0.250 | 0.250 | 0.500 | Synergy | 4 |
| AB08 | 8 | 128 | 1 | 32 | 0.125 | 0.250 | 0.375 | Synergy | 8 |
| AB09 | 8 | 128 | 0.5 | 64 | 0.063 | 0.500 | 0.563 | Additive | 16 |
| AB10 | 4 | 128 | 0.5 | 32 | 0.125 | 0.250 | 0.375 | Synergy | 8 |

*Selection rule: For each isolate, the concentration pair with the lowest ΣFICI (C_Comb_) was selected. If multiple pairs yielded the same ΣFICI, the pair with the lower colistin MIC in combination was chosen. Interaction categories were defined as: synergy (ΣFICI ≤ 0.5), additive (>0.5–<1), indifferent (≥1–<4), antagonism (≥4). Colistin MIC fold-change was calculated as Alone MIC_COL_/Combination MIC_COL_. MIC: Minimum inhibitory concentration; FIC: fractional inhibitory concentration; FICI: fractional inhibitory concentration index; COL: colistin; CIN: cinnamaldehyde.*

**Table S4.** Isolate-level checkerboard results for the colistin-eugenol combination against colistin-resistant *A. baumannii*.

| **Isolates** | **Alone MIC_COL_** | **Alone MIC_EUG_** | **Combination MIC_COL_** | **Combination MIC_EUG_** | **FIC_COL_** | **FIC_EUG_** | **∑FICI** | **Description** | **Colistin MIC fold-change** |
| --- | --- | --- | --- | --- | --- | --- | --- | --- | --- |
| AB01 | 4 | 512 | 2 | 32 | 0.500 | 0.063 | 0.563 | Additive | 2 |
| AB02 | 32 | 1024 | 2 | 512 | 0.063 | 0.500 | 0.563 | Additive | 16 |
| AB03 | 16 | 512 | 4 | 256 | 0.250 | 0.500 | 0.750 | Additive | 4 |
| AB04 | 16 | 512 | 8 | 32 | 0.500 | 0.063 | 0.563 | Additive | 2 |
| AB05 | 4 | 512 | 0.5 | 64 | 0.125 | 0.125 | 0.250 | Synergy | 8 |
| AB06 | 8 | 512 | 2 | 32 | 0.250 | 0.063 | 0.313 | Synergy | 4 |
| AB07 | 4 | 256 | 0.5 | 64 | 0.125 | 0.250 | 0.375 | Synergy | 8 |
| AB08 | 8 | 512 | 0.5 | 128 | 0.063 | 0.250 | 0.313 | Synergy | 16 |
| AB09 | 8 | 256 | 2 | 128 | 0.250 | 0.500 | 0.750 | Additive | 4 |
| AB10 | 4 | 512 | 1 | 32 | 0.250 | 0.063 | 0.313 | Synergy | 4 |

*Selection rule: For each isolate, the concentration pair with the lowest ΣFICI (C_Comb_) was selected. If multiple pairs yielded the same ΣFICI, the pair with the lower colistin MIC in combination was chosen. Interaction categories were defined as: synergy (ΣFICI ≤ 0.5), additive (>0.5–<1), indifferent (≥1–<4), antagonism (≥4). Colistin MIC fold-change was calculated as Alone MIC_COL_/Combination MIC_COL_. MIC: Minimum inhibitory concentration; FIC: fractional inhibitory concentration; FICI: fractional inhibitory concentration index; COL: colistin; EUG: eugenol.*

**Table S5.** Quantitative biofilm biomass and biofilm classification of colistin-resistant *A. baumannii.*

| **Isolate** | **OD_Mean_** | **SD** | **Biofilm degree** | **Comment** |
| --- | --- | --- | --- | --- |
| AB01 | 1.206 | 0.036 | III | Strong biofilm |
| AB02 | 0.695 | 0.107 | II | Moderate biofilm |
| AB03 | 1.240 | 0.052 | III | Strong biofilm |
| AB04 | 1.110 | 0.077 | II | Moderate biofilm |
| AB05 | 0.823 | 0.067 | II | Moderate biofilm |
| AB06 | 1.185 | 0.016 | III | Strong biofilm |
| AB07 | 0.932 | 0.059 | II | Moderate biofilm |
| AB08 | 1.082 | 0.020 | II | Moderate biofilm |
| AB09 | 0.671 | 0.052 | II | Moderate biofilm |
| AB10 | 1.053 | 0.088 | II | Moderate biofilm |
| Negative Control | 0.293 | 0.017 | - | - |

*Values are presented as mean OD_595_. Biofilm categories were assigned using negative control (OD_NC_); OD ≤ OD_NC_, non-biofilm producers (0); OD_NC_ < OD ≤ 2xOD_NC_, weak biofilm producers (I); 2xOD_NC_ < OD ≤ 4xOD_NC_, moderate biofilm producers (II); and OD > 4xOD_NC_, strong biofilm producers (III). OD: optical density, SD: standard deviation*

**Table S6.** Isolate-level biofilm inhibition by colistin-cinnamaldehyde and colistin-eugenol combinations in colistin-resistant *A. baumannii*.

| **Isolate** | **EOC** | **ID** | **COL (µg mL^-1^)** | **CIN (µg mL^-1^)** | **EUG (µg mL^-1^)** | **Mean_ODcorrected_ (±SD)** | **Biofilm remaining (%)** | **Biofilm inhibition (%)** |
| --- | --- | --- | --- | --- | --- | --- | --- | --- |
| AB04 | - | Positive Control | - | - | - | 1.540 (±0.094) | 100.00 | 0.00 |
| AB04 | CIN | 0.5xCOL_Comb_/0.5xCIN_Comb_ | 1 | 16 | - | 1.523 (±0.039) | 98.87 | 1.13 |
| AB04 | CIN | 0.5xCOL_Comb_/1xCIN_Comb_ | 1 | 32 | - | 1.439 (±0.062) | 93.39 | 6.61 |
| AB04 | CIN | 1xCOL_Comb_/0.5xCIN_Comb_ | 2 | 16 | - | 1.054 (±0.059) | 68.43 | 31.57 |
| AB05 | - | Positive Control | - | - | - | 1.569 (±0.144) | 100.00 | 0.00 |
| AB05 | CIN | 0.5xCOL_Comb_/0.5xCIN_Comb_ | 0.25 | 16 | - | 0.992 (±0.040) | 63.25 | 36.75 |
| AB05 | CIN | 0.5xCOL_Comb_/1xCIN_Comb_ | 0.25 | 32 | - | 0.916 (±0.021) | 58.37 | 41.63 |
| AB05 | CIN | 1xCOL_Comb_/0.5xCIN_Comb_ | 0.5 | 16 | - | 0.776 (±0.013) | 49.47 | 50.53 |
| AB05 | EUG | 0.5xCOL_Comb_/0.5xEUG_Comb_ | 0.25 | - | 32 | 1.186 (±0.058) | 75.61 | 24.39 |
| AB05 | EUG | 0.5xCOL_Comb_/1xEUG_Comb_ | 0.25 | - | 64 | 1.003 (±0.031) | 63.95 | 36.05 |
| AB05 | EUG | 1xCOL_Comb_/0.5xEUG_Comb_ | 0.5 | - | 32 | 0.916 (±0.066) | 58.37 | 41.63 |
| AB06 | - | Positive Control | - | - | - | 2.003 (±0.056) | 100.00 | 0.00 |
| AB06 | EUG | 0.5xCOL_Comb_/0.5xEUG_Comb_ | 1 | - | 16 | 1.519 (±0.067) | 75.81 | 24.19 |
| AB06 | EUG | 0.5xCOL_Comb_/1xEUG_Comb_ | 1 | - | 32 | 1.449 (±0.028) | 72.32 | 27.68 |
| AB06 | EUG | 1xCOL_Comb_/0.5xEUG_Comb_ | 2 | - | 16 | 0.401 (±0.006) | 19.99 | 80.01 |
| AB07 | - | Positive Control | - | - | - | 1.889 (±0.035) | 100.00 | 0.00 |
| AB07 | CIN | 0.5xCOL_Comb_/0.5xCIN_Comb_ | 0.5 | 8 | - | 1.668 (±0.071) | 88.30 | 11.70 |
| AB07 | CIN | 0.5xCOL_Comb_/1xCIN_Comb_ | 1 | 8 | - | 1.676 (±0.006) | 88.70 | 11.30 |
| AB07 | CIN | 1xCOL_Comb_/0.5xCIN_Comb_ | 0.5 | 16 | - | 1.521 (±0.042) | 80.52 | 19.48 |
| AB07 | EUG | 0.5xCOL_Comb_/0.5xEUG_Comb_ | 0.25 | - | 32 | 1.668 (±0.067) | 88.27 | 11.73 |
| AB07 | EUG | 0.5xCOL_Comb_/1xEUG_Comb_ | 0.5 | - | 32 | 1.477 (±0.022) | 78.16 | 21.84 |
| AB07 | EUG | 1xCOL_Comb_/0.5xEUG_Comb_ | 0.25 | - | 64 | 1.357 (±0.133) | 71.84 | 28.16 |
| AB08 | - | Positive Control | - | - | - | 1.734 (±0.099) | 100.00 | 0.00 |
| AB08 | CIN | 0.5xCOL_Comb_/0.5xCIN_Comb_ | 0.5 | 16 | - | 1.555 (±0.097) | 89.67 | 10.33 |
| AB08 | CIN | 0.5xCOL_Comb_/1xCIN_Comb_ | 0.5 | 32 | - | 1.511 (±0.151) | 87.18 | 12.82 |
| AB08 | CIN | 1xCOL_Comb_/0.5xCIN_Comb_ | 1 | 16 | - | 1.484 (±0.069) | 85.63 | 14.37 |
| AB08 | EUG | 0.5xCOL_Comb_/0.5xEUG_Comb_ | 0.25 | - | 64 | 1.262 (±0.083) | 72.76 | 27.24 |
| AB08 | EUG | 0.5xCOL_Comb_/1xEUG_Comb_ | 0.25 | - | 128 | 1.177 (±0.078) | 67.86 | 32.14 |
| AB08 | EUG | 1xCOL_Comb_/0.5xEUG_Comb_ | 0.5 | - | 64 | 1.035 (±0.016) | 59.70 | 40.30 |
| AB10 | - | Positive Control | - | - | - | 1.530 (±0.029) | 100.00 | 0.00 |
| AB10 | CIN | 0.5xCOL_Comb_/0.5xCIN_Comb_ | 0.25 | 16 | - | 1.363 (±0.097) | 89.09 | 10.91 |
| AB10 | CIN | 0.5xCOL_Comb_/1xCIN_Comb_ | 0.25 | 32 | - | 1.222 (±0.048) | 79.90 | 20.10 |
| AB10 | CIN | 1xCOL_Comb_/0.5xCIN_Comb_ | 0.5 | 16 | - | 0.957 (±0.101) | 62.54 | 37.46 |
| AB10 | EUG | 0.5xCOL_Comb_/0.5xEUG_Comb_ | 0.5 | - | 16 | 0.941 (±0.017) | 61.52 | 38.48 |
| AB10 | EUG | 0.5xCOL_Comb_/1xEUG_Comb_ | 0.5 | - | 32 | 0.891 (±0.027) | 58.25 | 41.75 |
| AB10 | EUG | 1xCOL_Comb_/0.5xEUG_Comb_ | 1 | - | 16 | 0.994 (±0.046) | 64.96 | 35.04 |

*Values are presented as mean OD595 (±SD), after background subtraction using the negative control wells (ODcorrected). “Positive Control” indicates biofilm growth without test agents. Combination ratios were defined relative to each isolate’s selected C_Comb_ (the concentration pair yielding the lowest ΣFICI in checkerboard testing). Abbreviations: COL: colistin; CIN: cinnamaldehyde; EUG: eugenol; OD: optical density.*

**Table S7.** Time-dependent biofilm remaining of colistin-cinnamaldehyde combination relative to PBS.

| **Isolates** | **Time (h)** | **Mean OD_PBS_** | **Mean**  **OD_COL-CIN_** | **Ratio**  **(COL-CIN/PBS)** | **−log10(Ratio)** | **% Remaining** |
| --- | --- | --- | --- | --- | --- | --- |
| AB04 | 1 | 0.9407 | 0.7747 | 0.8235 | 0.084 | 82.35% |
| AB04 | 4 | 0.9070 | 0.5612 | 0.6187 | 0.208 | 61.87% |
| AB04 | 8 | 0.6792 | 0.4137 | 0.6091 | 0.215 | 60.91% |
| AB04 | 24 | 0.6335 | 0.0815 | 0.1287 | 0.891 | 12.87% |
| AB05 | 1 | 0.6197 | 0.6063 | 0.9784 | 0.009 | 97.84% |
| AB05 | 4 | 0.5360 | 0.3762 | 0.7019 | 0.154 | 70.19% |
| AB05 | 8 | 0.5072 | 0.3167 | 0.6244 | 0.205 | 62.44% |
| AB05 | 24 | 0.4680 | 0.0760 | 0.1624 | 0.789 | 16.24% |
| AB07 | 1 | 0.7382 | 0.7083 | 0.9595 | 0.018 | 95.95% |
| AB07 | 4 | 0.7028 | 0.6238 | 0.8876 | 0.052 | 88.76% |
| AB07 | 8 | 0.5977 | 0.4442 | 0.7432 | 0.129 | 74.32% |
| AB07 | 24 | 0.7285 | 0.2528 | 0.3470 | 0.460 | 34.70% |
| AB08 | 1 | 1.4167 | 1.2002 | 0.8472 | 0.072 | 84.72% |
| AB08 | 4 | 1.2610 | 0.9332 | 0.7400 | 0.131 | 74.00% |
| AB08 | 8 | 1.2547 | 0.5743 | 0.4577 | 0.339 | 45.77% |
| AB08 | 24 | 1.2450 | 0.1475 | 0.1185 | 0.926 | 11.85% |
| AB10 | 1 | 0.8702 | 0.6787 | 0.7799 | 0.108 | 77.99% |
| AB10 | 4 | 0.7775 | 0.5832 | 0.7501 | 0.125 | 75.01% |
| AB10 | 8 | 0.5847 | 0.5142 | 0.8794 | 0.056 | 87.94% |
| AB10 | 24 | 0.7100 | 0.2165 | 0.3049 | 0.516 | 30.49% |

*Values are presented as mean OD595. Remaining (%) represents biofilm biomass in the treatment wells normalized to the PBS control at the same time point. Abbreviations: PBS: phosphate-buffered saline; OD: optical density; COL: colistin; CIN: cinnamaldehyde.*

**Table S8.** Time-dependent biofilm remaining of colistin-eugenol combination relative to PBS.

| **Isolates** | **Time (h)** | **Mean OD_PBS_** | **Mean**  **OD_COL-EUG_** | **Ratio**  **(COL-EUG/PBS)** | **−log10(Ratio)** | **% Remaining** |
| --- | --- | --- | --- | --- | --- | --- |
| AB05 | 1 | 0.6197 | 0.5173 | 0.8348 | 0.078 | 83.48% |
| AB05 | 4 | 0.5360 | 0.2855 | 0.5326 | 0.274 | 53.26% |
| AB05 | 8 | 0.5072 | 0.2987 | 0.5889 | 0.230 | 58.89% |
| AB05 | 24 | 0.4680 | 0.1008 | 0.2154 | 0.667 | 21.54% |
| AB06 | 1 | 0.9307 | 0.6747 | 0.7249 | 0.140 | 72.49% |
| AB06 | 4 | 0.7855 | 0.5352 | 0.6813 | 0.167 | 68.13% |
| AB06 | 8 | 0.7737 | 0.4553 | 0.5885 | 0.230 | 58.85% |
| AB06 | 24 | 1.0105 | 0.1458 | 0.1443 | 0.841 | 14.43% |
| AB07 | 1 | 0.7382 | 0.752 | 1.0187 | (-)0.008 | 101.87% |
| AB07 | 4 | 0.7028 | 0.6485 | 0.9227 | 0.035 | 92.27% |
| AB07 | 8 | 0.5977 | 0.5242 | 0.8770 | 0.057 | 87.70% |
| AB07 | 24 | 0.7285 | 0.1360 | 0.1867 | 0.729 | 18.67% |
| AB08 | 1 | 1.4167 | 1.0937 | 0.7720 | 0.112 | 77.20% |
| AB08 | 4 | 1.2610 | 0.8595 | 0.6816 | 0.167 | 68.16% |
| AB08 | 8 | 1.2547 | 0.5663 | 0.4513 | 0.346 | 45.13% |
| AB08 | 24 | 1.2450 | 0.0932 | 0.0749 | 1.126 | 7.49% |
| AB10 | 1 | 0.8702 | 0.6937 | 0.7972 | 0.098 | 79.72% |
| AB10 | 4 | 0.7775 | 0.5695 | 0.7325 | 0.135 | 73.25% |
| AB10 | 8 | 0.5847 | 0.5022 | 0.8589 | 0.066 | 85.89% |
| AB10 | 24 | 0.7100 | 0.1935 | 0.2725 | 0.565 | 27.25% |

*Values are presented as mean OD595. Remaining (%) represents biofilm biomass in the treatment wells normalized to the PBS control at the same time point. Abbreviations: PBS: phosphate-buffered saline; OD: optical density; COL: colistin; EUG: eugenol.*
